# Influence of dust deposition on optimal sizing of PV-battery system in arid region: a case study for Saudi Arabia

**DOI:** 10.1038/s41598-026-56634-y

**Published:** 2026-06-05

**Authors:** Ahmad Bilal Ahmadullah, Ahmed Amine Hachicha, Ahmad Shah Irshad, Tomonobu Senjyu

**Affiliations:** 1 Department of Energy Engineering, Engineering Faculty, Kandahar University, 3801 Kandahar, Afghanistan; 2 Sustainable and Renewable Energy Engineering Department, University of Sharjah, PO Box 27272, Sharjah, United Arab Emirates; 3 Sustainable Energy and Power System Research Centre, Research Institute for Science and Engineering, University of Sharjah, Sharjah, United Arab Emirates; 4 Department of Electrical and Electronics Engineering, Faculty of Engineering, University of the Ryukyus, Okinawa, 903-0213 Japan

**Keywords:** PV systems, Battery storage, Dust accumulation, SEM/EDS analysis, Optimal sizing, Energy science and technology, Engineering, Environmental sciences

## Abstract

Dust accumulation is a major challenge for PV systems in arid regions, reducing efficiency and increasing system costs. This study analyzes the influence of dust on the optimal sizing of a stand-alone PV-battery system for a typical household in Saudi Arabia. Dust samples were examined using SEM and EDS, showing irregular particles (1–100 µm, average ~ 50 µm) composed mainly of oxygen (58.6%) and silicon (11.8%). These particles reduced glass transmittance from 1 (clean) to 0.704, 0.496, and 0.349 at dust densities of 0.7, 1.4, and 2.1 mg/cm^2^, respectively. A MATLAB-based techno-economic model was developed to optimize system design under each condition while ensuring 0% loss of power supply probability. Under clean conditions, to meet a typical household load of approximately 2 kW, the optimal system required 10.5 kWp PV (21 modules) and 5 batteries at an LCOE of 0.22 $/kWh. At 0.7 mg/cm^2^, the requirement increased to 15 kWp PV (30 modules) with 5 batteries at 0.27 $/kWh. For 1.4 mg/cm^2^, 19.5 kWp PV (39 modules) and 6 batteries were needed, raising LCOE to 0.33 $/kWh. At 2.1 mg/cm^2^, the system required 21 kWp PV (42 modules) and 9 batteries, resulting in 0.40 $/kWh, an 82% cost increase. Results highlight the need for dust-mitigation strategies to ensure reliable and cost-effective PV system performance overall.

## Introduction

The global demand for electricity continues to increase due to population growth and economic development. While fossil fuels still dominate electricity generation, their environmental impacts have accelerated the transition toward renewable energy systems^1–3^. Among renewable technologies, photovoltaic (PV) systems have gained significant attention due to their scalability and suitability for high-solar-resource regions.

PV modules, typically composed of semiconductor materials such as silicon, convert solar radiation directly into electricity. When sunlight strikes the PV surface, photons excite electrons, producing electron–hole pairs that generate current^4^. Despite these advantages, the performance of PV modules is highly sensitive to external conditions, with dust accumulation on the module surface being a critical challenge^5^.

Dust deposition is particularly problematic in arid and semi-arid regions, where suspended particles frequently settle on PV surfaces. Depending on dust characteristics, environmental conditions, exposure duration, and module orientation, dust accumulation can reduce PV power output by up to 60%^6^. Dust particles scatter and absorb incident solar radiation, increase surface reflectivity, and create hotspots, which can lead to long-term material degradation. Such losses not only diminish efficiency but also compromise system reliability and economic viability^7,8^.

Saudi Arabia possesses one of the world’s highest solar energy potentials, with national average daily solar radiation typically reported in the range of 6.5–7.5 kWh/m^2^/day^9^. However, site-specific solar resources vary across regions depending on local climatic conditions. The country has rapidly expanded its PV sector, with an installed capacity of 2.3 GW in 2023 and ambitious plans to reach 58.7 GW by 2030^10^. However, frequent dust storms and arid climate conditions pose severe operational challenges for PV systems. Dust deposition on PV surfaces reduces energy production and can increase operational and maintenance requirements, thereby affecting the long-term economic performance of PV installations^11^. Addressing this issue is essential for ensuring the long-term success of PV systems in the region.

Numerous studies have investigated the effect of dust on PV performance under different climatic conditions. For instance, Kazem et al.^12^ reported a 35–40% reduction in performance over three months in Oman, while Dhaouadi et al.^13^ found a 30% transmittance reduction after four months of exposure in the UAE. In Europe, Radziemska^14^ observed a 3% annual performance reduction in Poland, whereas in Dhahran, a 5 g/m^2^ dust density caused a 20% decrease in transmittance within 45 days^15^. Similarly, Chen et al.^16^ found that dust deposition in China reduced PV power by 7.4% in a single week, with dust mainly composed of SiO₂ and CaCO₃. Tanesab et al.^17^ further demonstrated that performance degradation across seven PV technologies ranged between 19 and 33%. These studies highlight the global extent of the dust challenge but primarily emphasize performance degradation and cleaning strategies rather than system-level design implications.

In addition to these studies, numerous investigations across Saudi Arabia and other regions have reported significant dust-related impacts on PV performance, cleaning costs, and system sustainability. A summary of selected studies is provided in Table 1. It should be noted that the impact of dust on PV performance is highly location-dependent, influenced by regional climate, dust composition, particle size distribution, humidity, wind patterns, and cleaning practices.

**Table 1:** Studies addressing the impacts of dust on Solar PV device.

References	Year	Country	Main outcomes
Mehmood et al.^18^	2017	Saudi Arabia	The research examined the particle size distribution, morphology, and elemental composition of dust collected in Dhahran, assessing how these characteristics influence the performance of PV module transparent covers
Khan et al.^19^	2022	Saudi Arabia	The investigation evaluated the effectiveness of anti-soiling coatings under various climatic conditions in Saudi Arabia. The performance was influenced by factors such as site location, coating type, and exposure duration, with certain coatings achieving more than 30% reduction in soiling-related losses
AlZahrani^20^	2023	Saudi Arabia	The study reported a 40% decrease in average power output over a two-year period in Saudi Arabia and included an analysis of the physical and chemical characteristics of the dust
Khonkar et al.^21^	2014	Saudi Arabia	The study examined a 10 kW PV system after two years of operation and found that dust deposition had reduced its power output by approximately 20%
Herrmann et al.^22^	2015	Saudi Arabia	Findings indicated that PV efficiency losses could range between 30 and 80% under severe dust conditions
Benghanem et al.^23^	2018	Saudi Arabia	The study was conducted in Madinah city and reported a 28% loss in PV output power over a 60-day period due to dust deposition
Garni^24^	2022	Saudi Arabia	The research reviewed dust characterization in Saudi Arabia and reported the dust effects on the performance reduction of PV in various regions of Saudi Arabia, ranging from 2 to 50% depending on many factors. A case in Dhahran showed that the PV power was reduced by 50% in 6 months without cleaning
Baras et al.^25^	2013	Saudi Arabia	The annual cost of dust deposition, considering cleaning expenses and lost revenue was estimated to be 27.2 SAR/kW. With the application of machine-assisted cleaning technique, the cost is reduced to 11.2 SAR/kW
Sengupta et al.^26^	2023	India	A 10-kW rooftop PV system was investigated; the reported daily energy yield loss was 1.4%
Elamim et al.^27^	2023	Morrocco	The study reported that the PV current and power were reduced by 11.6–18% and 7.4–12.35% respectively due to dust deposition
Chanchangi et al.^28^	2020	Nigeria	This study reviews the impacts of dust deposition on PV performance and proposes dust cleaning techniques
Zhao et al.^29^	2021	China	It is found that when dust particle diameter decreases from 30 µm to 10 µm, the dust deposition is increased from 2.59 g/m^2^ to 9.76 g/m^2^, resulting PV efficiency reduction from 3.89% to 14.64%, and the transmittance was reduced from 25.32% to 8.38%
Chen et al.^16^	2020	China	It is reported that the dust deposition density on PV modules in China is 0.644 g/m^2^ in a week and the dust decreased the PV power by 7.4% weekly
Hachicha et al.^30^	2019	UAE	The experiments conducted in a 5-month period indicated 12.7% soiling loss corresponding to 5.44 g/m^2^ dust deposition density. Dust deposition depends on tilt angle, the dust deposition density increased by 37.63%, 14.11%, and 10.95% at 0°, 25°, and 45° tilt angles respectively
Ahmed et al.^31^	2023	Bangladesh	It is reported that the presence of dust on the PV surface reduced power from 46.81W to 21W, and 5 m/s wind velocity increased performance from 14.8% to 16.5%

While the studies summarized in Table 1 provide valuable insights into dust characteristics, performance degradation, and mitigation strategies, they primarily emphasize module-level impacts. What remains underexplored is the impact of dust on the optimal sizing of PV-battery systems under realistic operating constraints^32^. Since dust reduces the energy harvested from PV modules, it directly affects the system’s ability to meet demand with available generation. This increases the dependence on battery storage and may require additional PV capacity to maintain full reliability. Neglecting dust effects in system design can therefore lead to underestimated storage requirements, oversizing after installation, and higher LCOE.

To address this gap, the present study investigates how dust accumulation influences the optimal sizing of stand-alone PV arrays and battery storage systems for a typical residential house in Saudi Arabia. Two scenarios are considered: (i) a clean PV surface, and (ii) dusty surfaces with deposition densities of 0.7, 1.4, and 2.1 mg/cm^2^, selected to represent low, moderate, and severe soiling conditions based on experimentally characterized dust properties and reported regional accumulation data (see "Dust analysis using SEM/EDS" section and Fig. 3). In all cases, the system is designed to meet the entire household load with 100% renewable energy contribution and a loss of power supply probability of 0%. By comparing clean and dusty conditions, the study quantifies how dust-driven performance degradation propagates to increased PV capacity and battery size requirements. The findings provide practical insights for PV-battery system designers, investors, and policymakers in arid regions to account for dust-related effects in techno-economic assessments and to optimize system sustainability.

It should be emphasized that the different PV capacities obtained under varying dust conditions do not represent real-time adjustments of a single system. In practical applications, PV system capacity is fixed after installation and cannot be modified based on changing environmental conditions. The configurations presented in this study correspond to independent design scenarios, intended to evaluate how different levels of dust accumulation influence the optimal sizing of PV-battery systems during the planning stage.

## Contributions

Dust accumulation is one of the most persistent challenges for PV systems in arid regions, where frequent deposition significantly reduces energy yield and compromises system economics. While past studies have primarily focused on performance degradation and cleaning methods, there is a lack of research on how dust affects the techno-economic optimization of PV-battery systems^32^. This study addresses this gap by investigating the extent to which dust increases the required PV capacity and battery storage to achieve reliable household electrification. By explicitly integrating dust effects into the optimization process, the work provides insights into both technical design and economic feasibility. The novelty of the present work lies in linking experimentally characterized dust properties directly with reliability-based techno-economic optimization of PV-battery system sizing and cost evaluation, which has received limited attention in previous studies.

The key contributions of this study are as follows:

- Experimental dust characterization: Performed morphological and compositional analysis of locally collected dust (SEM and EDS) to derive realistic optical parameters used in the PV performance and optimization model.
- Dust-aware optimization framework: Developed a sizing model that incorporates dust deposition levels (0.7, 1.4, and 2.1 mg/cm^2^) into PV-battery system design.
- Clean vs. dusty comparison: Quantified how PV capacity and battery size increase under dusty conditions relative to clean surfaces.
- Reliability and sustainability constraints: Designed all systems to ensure 0% loss of power supply probability (LPSP) and 100% renewable energy contribution, guaranteeing full load coverage.
- Economic performance metric: Identified the optimal configuration as the one with the minimum LCOE, enabling direct comparison of clean and dusty scenarios.
- Residential case study in Saudi Arabia: Applied the framework to a typical household in an arid climate, offering regionally relevant insights for system designers and policymakers.
- Policy and investment guidance: Demonstrated how ignoring dust leads to underestimated system sizes and costs, underscoring the need for dust considerations in both local and national solar energy planning.

## Methodology

### Dust analysis using SEM/EDS

Scanning Electron Microscopy (SEM) combined with Energy Dispersive Spectroscopy (EDS) was performed on dust samples collected from the PV module surfaces. The objective of these analyses was to characterize the dust in terms of particle size distribution, surface morphology, and elemental composition. These characteristics are essential for understanding how dust influences light transmittance and overall PV performance.

Dust samples were collected from the surfaces of PV modules installed in the study area. The dust corresponded to naturally deposited environmental particles accumulated under outdoor operating conditions. The samples were gently removed using a clean soft brush, stored in sealed containers to avoid contamination, and dried under ambient laboratory conditions prior to SEM/EDS analysis. The size of the dust particles plays a critical role in determining the extent of shading losses, which directly reduces the short circuit current and therefore degrades PV energy generation. Smaller particles tend to pack more densely with fewer voids, thereby blocking a larger fraction of incident sunlight compared to larger particles^33^. Moreover, fine particles are distributed more uniformly across the surface and cause stronger scattering of short-wavelength light, further lowering transmittance. Under humid conditions, dust accumulation becomes even more problematic as moisture promotes the formation of a sticky layer, increasing particle adhesion and making removal more difficult^34^.

**Fig. 1:**
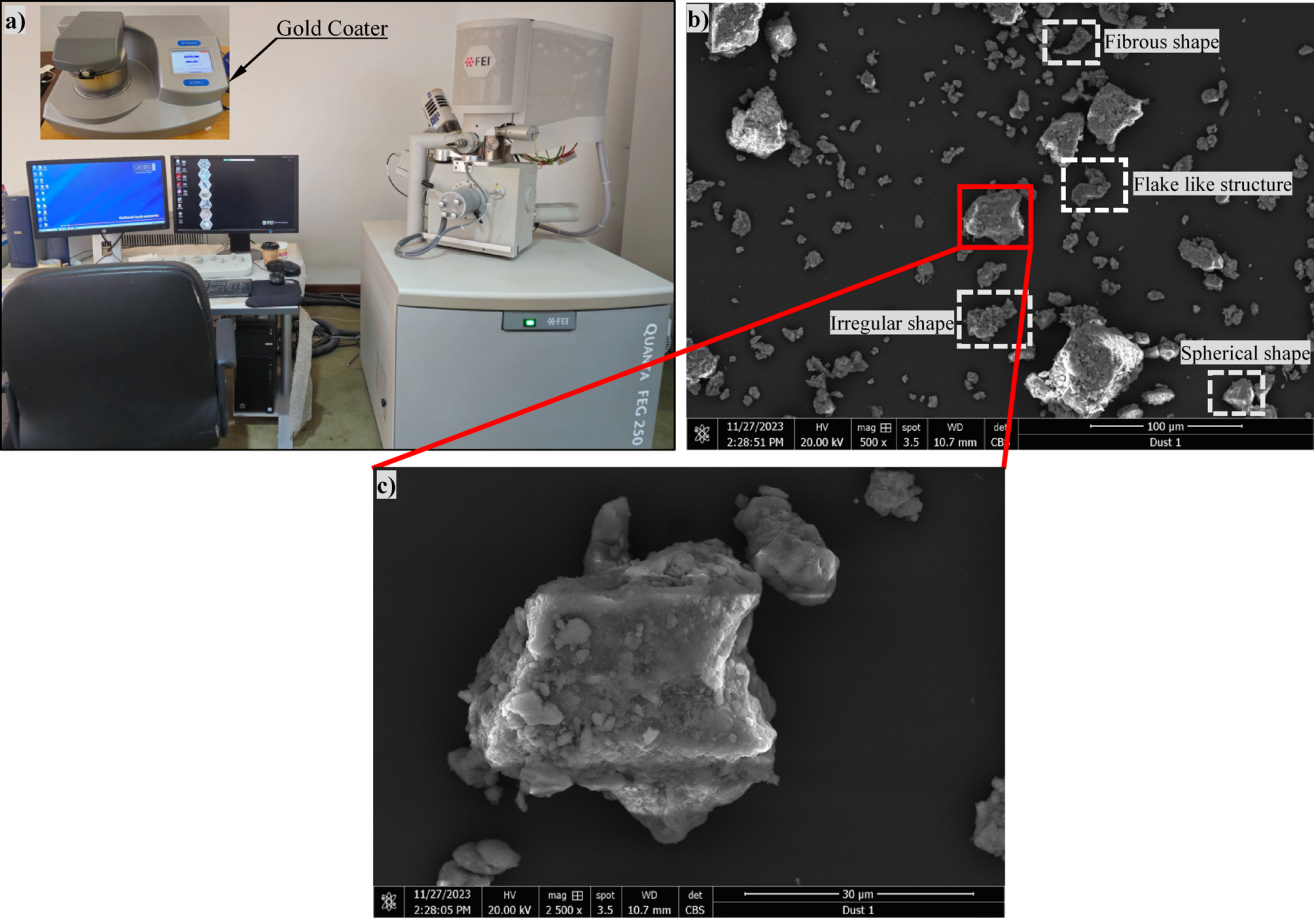
SEM of dust samples: (**a**) experimental setup with gold coater and QUANTA FEG-250 SEM, (**b**) SEM image showing dust particles of various shapes (spherical, irregular, fibrous, flake-like) and size distribution, and (**c**) magnified view of a selected irregular particle with smaller fragments attached to its surface due to electrostatic interactions.

Before imaging, around 5 mg of dust was placed on a polycarbonate substrate and coated with a thin layer of gold to enhance surface conductivity. The polycarbonate substrate was selected as a stable and inert support material for mounting loose dust particles during SEM preparation, allowing reliable imaging after gold coating. The samples were then analyzed using a QUANTA FEG-250 SEM (Fig. 1a)^35^, which provided detailed, high-resolution images of the dust morphology.

As illustrated in Fig. 1b, the particles displayed considerable variation in both size and distribution, with forms ranging from spherical with smooth surfaces to irregular grains with rough textures, fibrous structures, flake-like particles, and fragments with sharp edges or rounded corners. The observed particle sizes ranged from a few micrometers up to approximately 100 µm. Based on SEM visual inspection, a representative particle size of approximately 50 µm was adopted for subsequent modeling to characterize typical dust behavior. In certain regions, dust tended to cluster together, while in others it was more evenly dispersed. A closer view in Fig. 1c shows an irregularly shaped particle, with smaller fragments adhering to its surface, likely due to electrostatic attraction.

**Fig. 2:**
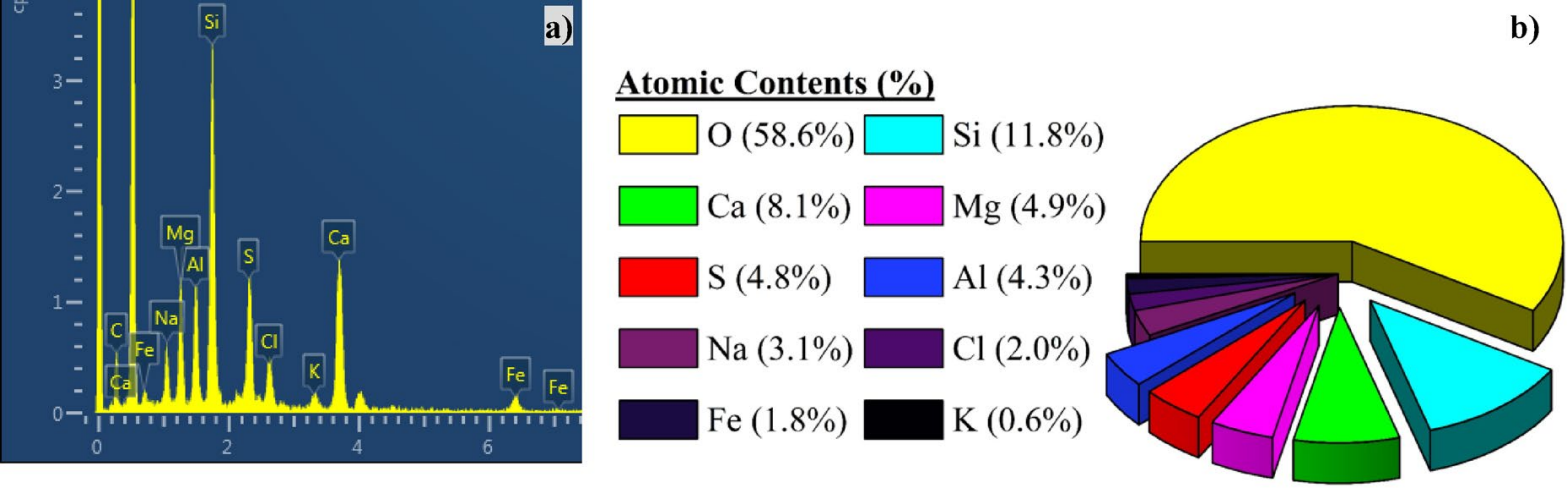
EDS analysis of dust sample: (**a**) EDS spectrum showing elemental peaks corresponding to O, Si, Ca, Mg, Al, S, Na, Cl, Fe, and K, and (**b**) quantitative atomic composition presented as a pie chart.

Figure 2 shows the EDS analysis of the collected dust sample. The spectrum (Fig. 2a) confirms the presence of several major and minor elements, with distinct peaks corresponding to O, Si, Ca, Mg, Al, S, Na, Cl, Fe, and K. Among these, oxygen exhibits the most prominent peak, highlighting its dominance in the dust composition. The quantitative analysis (pie chart) indicates that the dust is primarily composed of oxygen (58.6%) and silicon (11.8%), followed by calcium (8.1%), magnesium (4.9%), sulfur (4.8%), and aluminum (4.3%). Minor constituents include sodium (3.1%), chlorine (2.0%), iron (1.8%), and potassium (0.6%). These results demonstrate that the dust is largely made up of oxides and silicate compounds, which are known to strongly influence PV surface soiling and transmittance reduction.

**Fig. 3:**
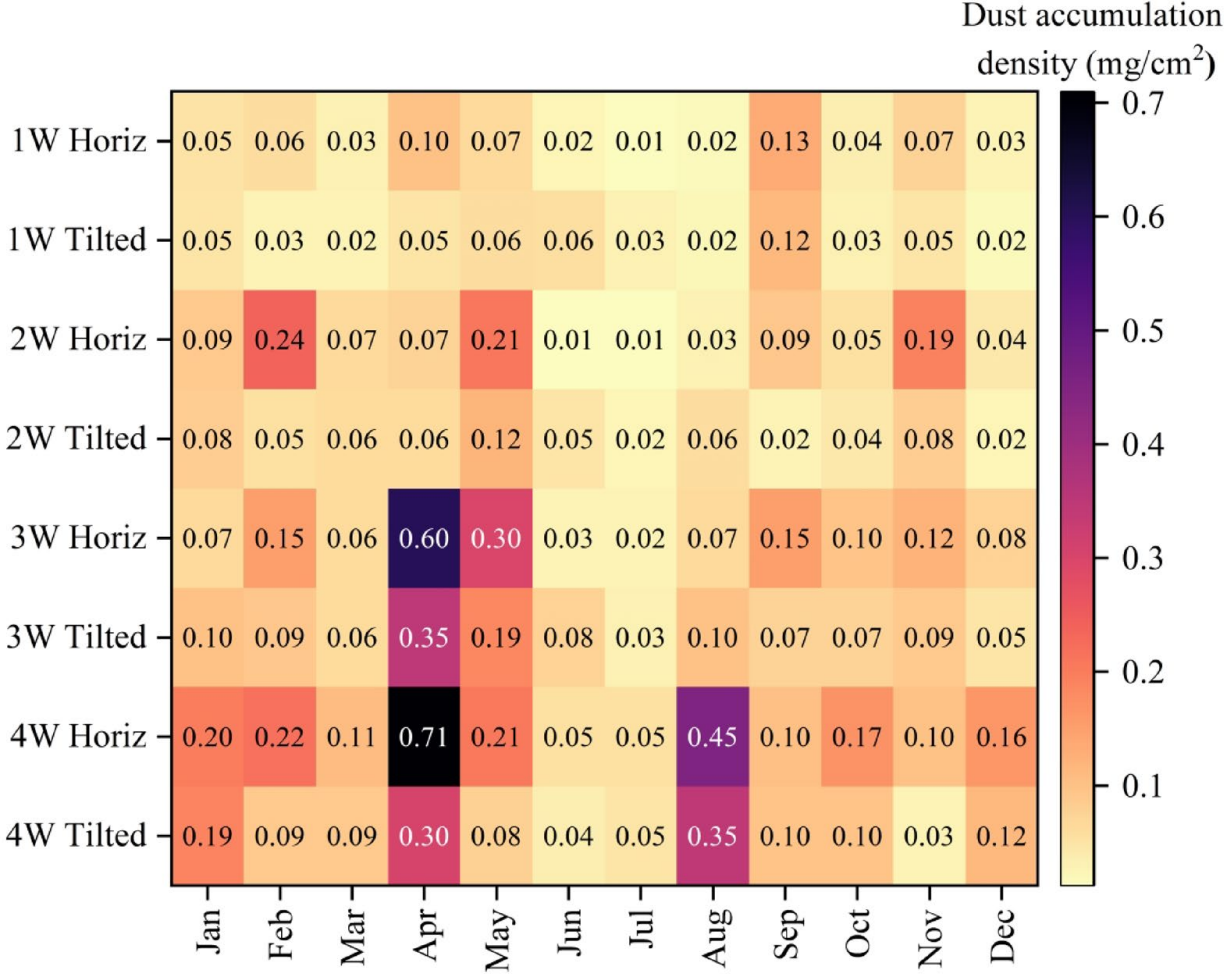
Monthly dust accumulation density (mg/cm^2^) on horizontal and tilted glass surfaces at a tilt angle of 15° for 1–4 weeks of exposure^36^.

The dust accumulation densities used in this study were selected based on environmental conditions typical of Saudi Arabia, where frequent dust storms contribute to substantial deposition on exposed surfaces. Such deposits significantly reduce the transmittance of PV modules, thereby lowering the available energy for electricity generation. Previous research conducted in the Rumah area of Saudi Arabia reported monthly variations in dust accumulation on horizontal and tilted glass surfaces. Figure 3 illustrates these values for different exposure durations (1–4 weeks) throughout the year^36^. Based on the reported monthly accumulation data in Ref.^36^, the averaged dust deposition under extended exposure conditions approaches approximately 2.1 mg/cm^2^. This value was therefore adopted as a representative upper-bound soiling scenario in the present study. Although the present case study focuses on Dhahran, this value was considered representative of severe soiling conditions in the region. It should be noted that atmospheric particulate matter concentrations (e.g., PM2.5 and PM10) represent suspended dust in air and are not directly equivalent to deposited dust density on PV surfaces. Surface accumulation depends on additional environmental factors such as exposure duration, wind, and surface orientation.

Moreover, the conclusions of this study are based on the relative trends of increasing dust accumulation on PV-battery system sizing and LCOE, rather than on exact site-specific dust values. To realistically capture dust impacts under varying conditions, three accumulation densities, χ = 0.7, 1.4, and 2.1 mg/cm^2^, were selected for the optimization process, in addition to the clean-surface baseline (χ = 0 mg/cm^2^). These values represent low, moderate, and high dust scenarios, respectively, allowing assessment of how dust accumulations alter PV performance, required PV capacity, and battery storage sizing. The selected densities are derived from the range of dust deposition values reported under different exposure durations and orientations, as illustrated in Fig. 3, which serves as the reference basis for defining the representative soiling scenarios used in the optimization analysis. The particle size distribution obtained from SEM analysis was used to define the lower and upper limits of the dust particle radius ${R}_{d}$ employed in the transmittance model ("Transmittance reduction due to dust accumulation" section). Therefore, the experimentally characterized dust properties directly influence the estimation of optical losses and the resulting PV-battery system sizing.

### Transmittance reduction due to dust accumulation

Equation (1) is adapted from the NASA dust occlusion model, which describes the reduction of light transmission due to dust particles. In this study, the model is applied to PV glass surfaces to estimate how accumulated dust reduces optical transmittance. The equation provides an exponential relationship between dust accumulation and surface transmittance^37^.

1 $$\frac{{\tau}_{dusty}}{{\tau}_{clean}}=exp\left[\frac{-3\gamma {M}_{tot}}{4A\rho }\int \frac{3}{16{R}_{d}^{2}}d{R}_{d}\right]$$

where ${M}_{tot}$ is the accumulated dust mass, *A* is the surface area, *ρ* is the dust particle density, ${R}_{d}$ is the dust particle radius, and *γ* represents the relative optical transmittance of an individual dust particle. The equation relates the transmittance of dusty surfaces (${\tau}_{dusty}$) to that of a clean surface (${\tau}_{clean}$), accounting for deposited dust mass, particle-size distribution, particle density, and the optical attenuation characteristics of individual dust particles. The exponential term represents the cumulative optical extinction caused by dust particles, where optical extinction refers to the combined effects of light absorption and scattering by dust, which reduce the amount of solar radiation transmitted through the PV glass surface.

The parameter γ represents the relative optical transmittance of an individual dust particle, as defined in the NASA dust occlusion model, and should not be confused with τdusty, which describes the overall transmittance of the dust-covered PV surface. In this study, ${\tau}_{clean}$ was assumed to be equal to 1, while the dust particle radius was estimated from SEM observations. Based on SEM images showing particle diameters up to nearly 100 µm with representative sizes around 50 µm, an effective particle radius range of ${R}_{d}=0.5-25$ µm was adopted as the integration limits in Eq. (1), representing the dominant particle sizes contributing to optical attenuation. The parameter *γ* was taken as 0.45, and the dust particle density (*ρ*) was considered 2.47 g/cm^3^, consistent with values reported in previous literature^38^.

**Fig. 4:**
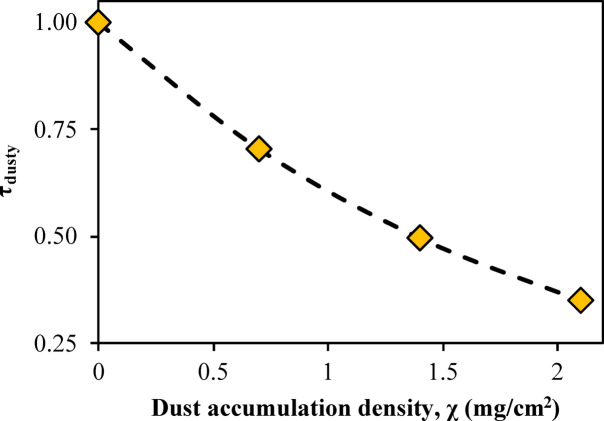
Transmittance (τ_dusty_) as a function of dust accumulation density (χ), showing a nonlinear decrease with increasing dust deposition.

Using these parameters, τ_dusty_ was calculated for different dust accumulation densities (χ = 0.7, 1.4, and 2.1 mg/cm^2^). The results are illustrated in Fig. 4. As expected, transmittance decreases nonlinearly with increasing dust density, showing an exponential decay trend. At a deposition of 0.7 mg/cm^2^, τ_dusty_ falls to 0.704, while higher densities of 1.4 mg/cm^2^ and 2.1 mg/cm^2^ further reduce transmittance to 0.496 and 0.349, respectively. These results clearly indicate that even moderate dust deposition can reduce light transmission by more than 50%, significantly lowering the effective irradiance reaching the PV surface.

This relationship highlights the critical role of dust accumulation in degrading PV performance. By integrating these calculated τ_dusty_ values into the PV-battery system model, the study quantifies the increase in required PV capacity and battery storage needed to maintain full load coverage under dusty conditions.

### System design and modeling

Mathematical models were developed to represent the performance of the PV-battery system under both clean and dusty surface conditions. The modeling framework incorporates system description, meteorological data, solar radiation estimation, PV module performance with dust effects, battery storage model, economic evaluation based on LCOE, system reliability criteria, and the applied sizing and optimization methodology.

#### Weather data and electric load profile

The performance of the PV-battery system was analyzed for Dhahran, located in the eastern province of Saudi Arabia at 26.23° N latitude and 50.03° E longitude. The city lies at an altitude of 17 m above sea level and covers an area of about 58.27 km^2^^39^. Dhahran is characterized by a hot desert climate with frequent dust storms and high wind activity, often reducing visibility to only a few meters. Its proximity to the Arabian Gulf also results in persistently high humidity levels, particularly in spring, summer, and autumn. The combination of dust deposition and moisture leads to the formation of sticky layers and mud on exposed PV surfaces, exacerbating soiling losses^39^.

**Fig. 5:**
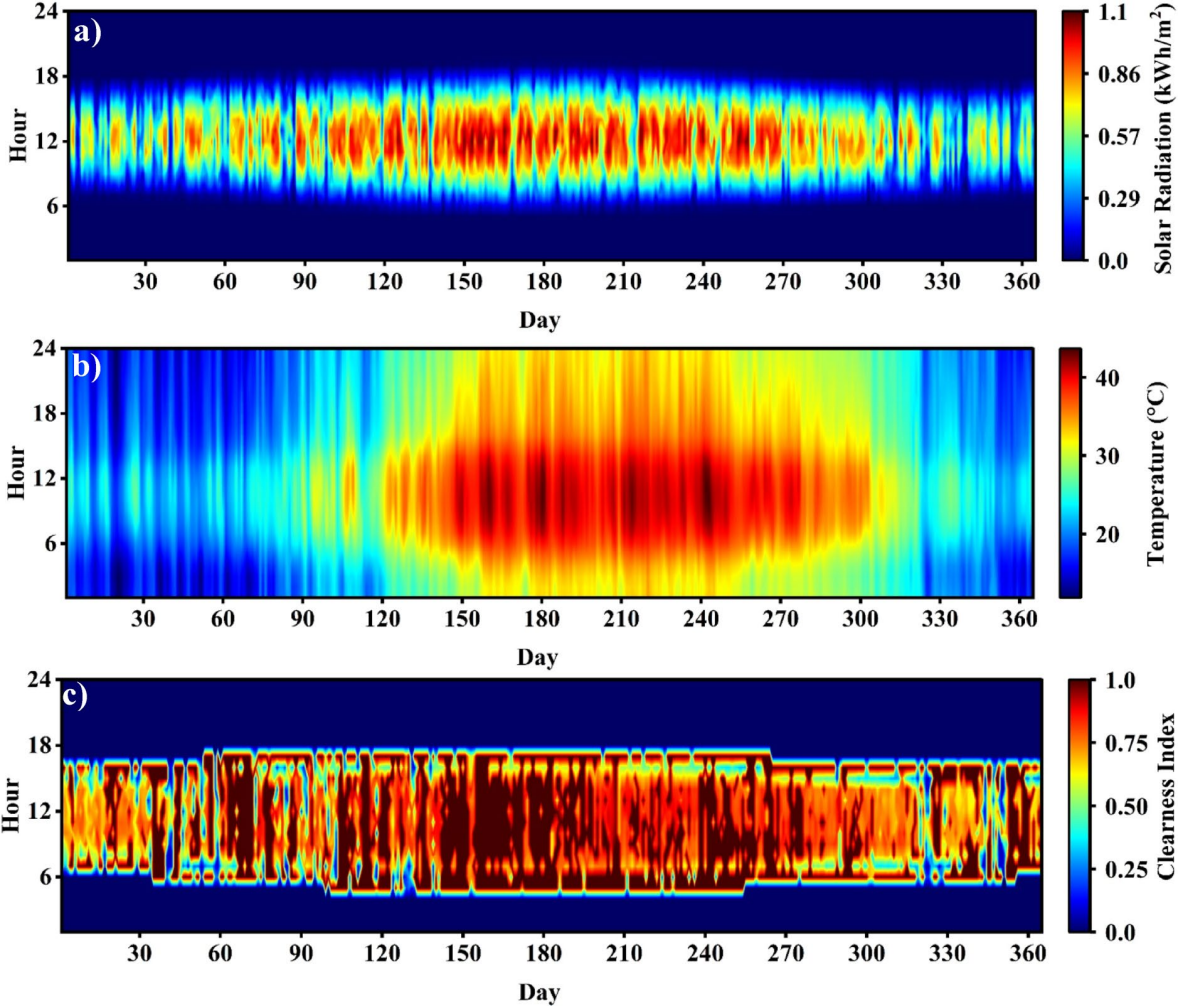
Hourly weather data for Dhahran, Saudi Arabia (Latitude: 26.23°N, Longitude: 50.03°E) from NREL^40^: (**a**) solar radiation on horizontal surface, (**b**) ambient temperature, and (**c**) clearness index.

For this study, hourly weather data from the National Renewable Energy Laboratory (NREL) was used to drive the simulations^40^. The variation of solar radiation, ambient temperature, and clearness index throughout the year is illustrated in Fig. 5. As shown in Fig. 5a, horizontal global solar radiation reaches its highest values during April to August, with peak hourly intensities approaching 1.1 kWh/m^2^. The annual average daily solar radiation is 5.05 kWh/m^2^/day. Figure 5b shows that the ambient temperature exhibits a strong seasonal cycle, with summer months frequently exceeding 40 °C, while the annual average remains around 27 °C. Finally, Fig. 5c presents the clearness index, which reflects atmospheric transparency and cloudiness. The index demonstrates considerable variability across the year, with lower values during dusty and humid periods, highlighting the strong influence of local environmental conditions on solar resource availability.

**Fig. 6:**
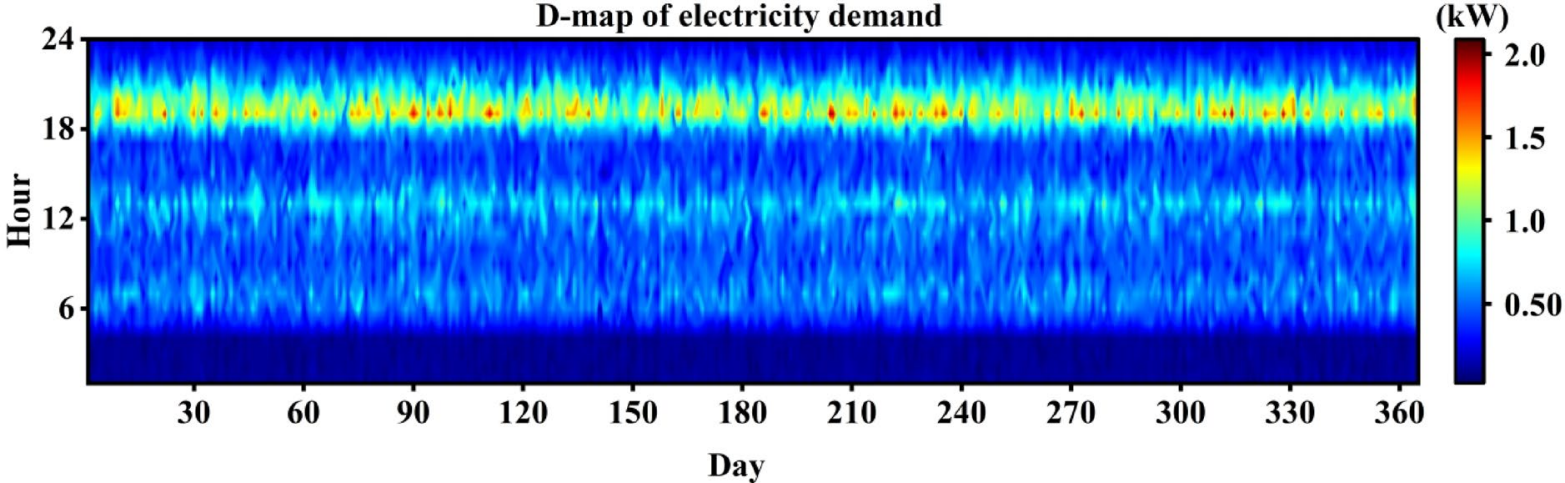
Hourly D-map of electricity demand for a typical household in Saudi Arabia.

Figure 6 illustrates the demand map (D-map) of the annual load profile for a typical house on an hourly basis. The typical residential electric load profile was taken from the standard dataset available in HOMER Pro software, which provides representative hourly demand patterns for residential systems. The highest load consistently occurs in the late afternoon and evening hours, with peak values approaching 2 kW, whereas demand during nighttime and early morning hours remains comparatively low.

#### System description

**Fig. 7:**
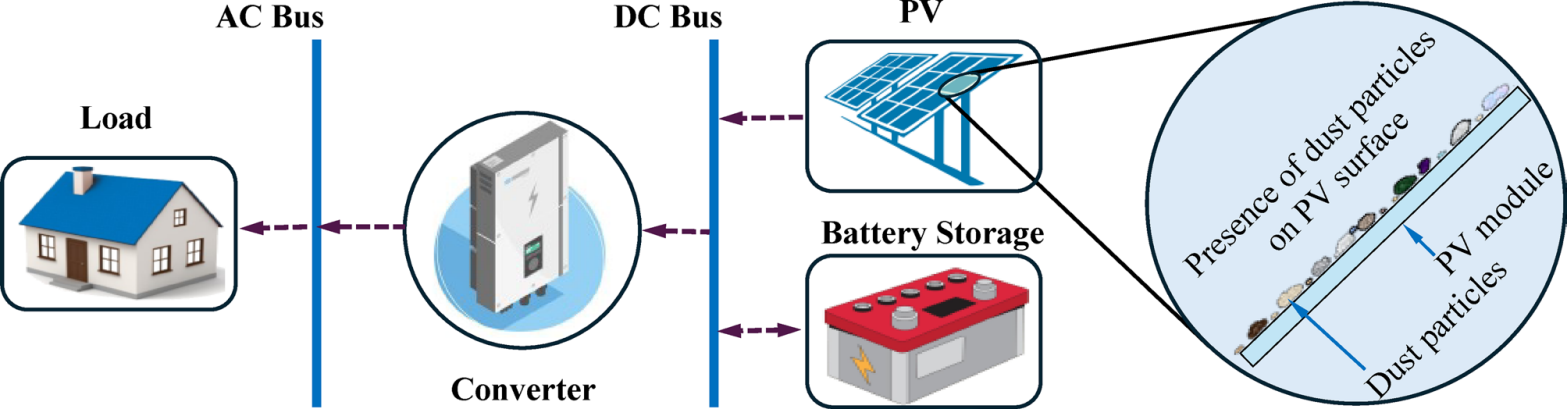
Schematic diagram of the PV-battery system showing the presence of dust particles on active PV module surfaces.

In this study, a stand-alone (off-grid) PV-battery system was modeled to evaluate the impact of dust accumulation on optimal system sizing (Fig. 7). The system consists of PV modules as the primary energy source, a battery bank for energy storage, and an inverter for AC/DC conversion, supplying power to a typical residential household load. Dust particles deposited on the active surface of the PV modules reduce the effective solar irradiance reaching the cells, thereby lowering system efficiency and increasing the required PV and battery capacities to ensure reliable power delivery.

The schematic diagram (Fig. 7) illustrates the interaction among system components: PV modules generate electricity under varying dust conditions, which is either stored in the battery bank or supplied directly to the household through the inverter. To maintain reliability, the system is designed to meet 100% of the electrical demand with a loss of power supply probability (LPSP) of 0%.

#### Solar radiation model on a tilted surface

To estimate the solar radiation incident on a tilted plane (I_T_), the Liu and Jordan isotropic sky model was applied^41^:

2 $${I}_{T}={I}_{b}{R}_{b}+\frac{{I}_{d}\left(1+\mathit{cos}\beta \right)}{2}+\frac{I{\rho}_{g}\left(1-\mathit{cos}\beta \right)}{2}$$

Here, I, I_b_, and I_d_ are the global, beam, and diffuse components of solar radiation on a horizontal surface, respectively. The parameter β denotes the tilt angle of the surface, and ρ_g_ is the ground reflectance, typically set to 0.2 for non-snow-covered surfaces.

The beam and diffuse radiation are expressed as^42^:

3 $${I}_{b}={I-I}_{d}$$

4 $$\frac{{I}_{d}}{I}=\left\{\begin{array}{l}1-0.09{k}_{T} for {k}_{T}\le 0.22\\ 0.9511-0.1604{k}_{T}+4.388{{k}_{T}}^{2}\\ -16.638{{k}_{T}}^{3}+12.336{{k}_{T}}^{4} for 0.22<{k}_{T}\le 0.8\\ 0.165 for {k}_{T}>0.8\end{array}\right.$$

The clearness index, k_T_​, is defined as the ratio of measured global radiation to extraterrestrial radiation^42^:

5 $${k}_{T}=\frac{I}{{I}_{o}}$$

The hourly extraterrestrial radiation, I_o_, is calculated as:

6 $${I}_{o}=\frac{12\times 3600}{\pi }{G}_{sc}\left(1+0.033\mathit{cos}\frac{360n}{365}\right)\times \left[\mathit{cos}\phi \mathit{cos}\delta \left(\mathit{sin}{\omega}_{2}-\mathrm{sin}{\omega}_{1}\right)+\frac{\pi \left({\omega}_{2}-{\omega}_{1}\right)}{180}\mathit{sin}\phi \mathit{sin}\delta \right]$$

where G_sc_ is the solar constant (1367 W/m^2^), n is the day of the year, ω_1_ and ω_2_ are the hour angles for the selected hour, and ϕ is the site latitude. The solar declination angle δ is determined as^42^:

7 $$\delta =23.45\mathit{sin}\left(360\frac{284+n}{365}\right)$$

The geometric factor, R_b_, representing the ratio of beam radiation on a tilted surface to that on a horizontal surface, is given by^43^:

8 $${R}_{b}=\frac{\mathit{cos}\left(\phi -\beta \right)\mathit{cos}\delta \mathit{cos}\omega +\mathit{sin}\left(\phi -\beta \right)\mathit{sin}\delta }{\mathit{cos}\phi \mathit{cos}\delta \mathit{cos}\omega +\mathit{sin}\phi \mathit{sin}\delta }$$

Here, the hour angle, ω, represents solar time in angular form, with 15∘ corresponding to one hour.

#### PV array model with the presence of the dust particles

In this study, a monocrystalline PV module with a nominal output power of 500 W_p_ and a surface area of 2.209 m^2^ was used to simulate the electrical energy production under clean and dusty surface conditions. To maximize solar energy capture, the PV module was assumed to be south-oriented and installed at a tilt angle equal to the site latitude (26.23°) without shading throughout the year. These assumptions represent standard best-practice installation conditions for residential PV systems in Saudi Arabia. They were adopted to isolate the impact of dust accumulation on PV-battery system sizing, rather than to assess the effects of orientation, tilt optimization, or shading. The influence of dust accumulation on the monocrystalline module was then incorporated into the simulation framework. The technical specifications of the PV module are presented in Table 2, while the economic parameters used in the optimization are summarized in Table 3.

**Table 2:** PV module technical specifications at STC^44^.

Characteristics	Value
Nominal power (P_max_)	500 W_p_
Opt. Operating Voltage (V_mp_)	45 V
Opt. Operating Current (I_mp_)	11.12 A
Open Circuit Voltage (V_oc_)	53.7 V
Short Circuit Current (I_sc_)	11.77 A
Module Efficiency	21.2%
Surface area	2.21 m^2^^45^
Temperature coefficient	− 0.0034/°C (equivalent to − 0.34%/°C)^45^
Module temperature at standard test conditions	25 °C^45^
Nominal operating cell temperature	46 °C^45^
Lifetime	25 years

**Table 3:** Economic parameters of the PV module^46^.

Parameter	Value
Initial cost (PV modules)	1100 $/kW
Replacement cost (PV modules)	1100 $/kW
Operation and maintenance	20 $/year

It should be noted that dust deposition is influenced by multiple environmental factors that cannot be completely represented in the model. However, its overall impact on system performance is primarily through surface shading and light scattering, which reduce the optical transmittance of the glass cover. This effect is represented by the soiling transmittance factor (τ_dusty_), which quantifies the reduction in transmitted radiation due to accumulated dust.

Empirical approaches have been widely adopted to estimate PV performance under varying conditions of solar irradiance, temperature, and dust deposition^47–49^. In this study, the following relationship was used to estimate the electrical efficiency of the PV module^50^:

9 $${\eta}_{el}={\eta}_{ref}\left[1-{\beta}_{ref}\left({T}_{cell}-298.15\right)\right]{\tau}_{dusty}$$

In this model, η_ref_ represents the module efficiency under standard test conditions (STC), while β_ref_ denotes the normalized temperature coefficient of maximum power of the PV module, expressed in fractional form per degree Celsius. In this study, βref is taken as − 0.0034 1/°C, which is equivalent to the commonly reported value of − 0.34%/°C for commercial monocrystalline PV modules. The parameter T_cell_ corresponds to the operating temperature of the PV cell, and τ_dusty_ is defined as the transmittance ratio between a dusty glass surface and a clean glass surface, accounting for the reduction in solar radiation due to dust accumulation.

The output power of the PV module under dust conditions is expressed as^50^:

10 $$P={\eta}_{el}{I}_{T}A{\tau}_{g}{\alpha}_{cell}$$

In this expression, A is the surface area of the PV module, the parameter τ_g_ denotes the transmissivity of the glass cover^51^, and α_cell_ corresponds to the absorptivity of the PV cells which is considered 0.94^52^, both of which determine the fraction of incoming solar radiation effectively absorbed and converted into electrical energy. I_T_ is the incident radiation on tilted PV surface, obtained from the aforementioned solar radiation model.

#### Converter model

The converter was modeled to account for the conversion of DC electricity from the PV array and battery bank into AC power for household use. In the adopted stand-alone PV-battery configuration, the converter operates as a hybrid unit that manages power flow between the PV system, battery storage, and household load. PV generation is first used to satisfy the load demand, while any surplus energy is directed to battery charging. The DC power required to supply the household load at any hour is expressed in terms of the converter efficiency (η_c_) as:

11 $${P}_{Demand}\left(t\right)=\frac{{P}_{L}\left(t\right)}{{\eta}_{c}}$$

where ${P}_{L}(t)$ is the household load demand at hour *t*, and η_c_ is the converter efficiency. In this study, the converter efficiency was taken as 95%^45^, consistent with values reported for residential-scale systems.

The converter rating was determined based on the peak household demand rather than the installed PV capacity. Since the electrical load remains the same for all dust scenarios, the converter size also remains unchanged. Increasing dust accumulation reduces PV output; therefore, additional PV modules are required to maintain annual energy adequacy, but this does not increase the instantaneous load demand handled by the converter. Surplus PV power is first used for battery charging. When the battery reaches its maximum state of charge, excess PV generation is regulated by the charge controller with maximum power point tracking (MPPT) functionality. In such cases, the controller limits the power extracted from the PV array by adjusting its operating point away from the maximum power point, thereby effectively reducing the delivered power. Consequently, the installed PV capacity may exceed the converter rating in stand-alone PV-battery systems because system sizing is governed by annual energy adequacy and reliability requirements rather than instantaneous load magnitude. The converter specifications are listed in Table 4.

**Table 4:** Converter specification^45,53^.

Parameter	Value	Unit
Rated power	2.2	kW
DC input voltage	48	V
AC output voltage	230	V
Efficiency	95	%
Capital cost	650	$/kW
Replacement cost	600	$/kW
Lifetime	15	year

#### Battery system model

In this study, the battery bank was modeled to evaluate the impact of dust-induced PV performance reduction on energy storage requirements and overall system economics. The total hourly power output from the PV array, P_PV_(t), was calculated based on the number of installed PV modules and their performance under varying dust conditions. The battery state of charge (SOC) at each hour was updated depending on the balance between PV generation and load demand. When net PV power was greater than the load, the surplus was stored in the battery after accounting for charging efficiency (η_chg_)^54^:

12 $$SOC\left(t+1\right)=\mathit{min}[SOC\left(t\right)+\left({P}_{PV}\left(t\right)-{P}_{Demand}\left(t\right)\right){\eta}_{chg},{SOC}_{max})]$$

Conversely, when demand exceeded PV generation, the battery discharged to supply the deficit, limited by the minimum allowable SOC defined by the depth of discharge (DOD)^46^:

13 $$SOC\left(t+1\right)=max[SOC\left(t\right)-\frac{{P}_{Demand}\left(t\right)-{P}_{PV}\left(t\right)}{{\eta}_{dis}}, {SOC}_{min}]$$

where $SO{C}_{min}=\left(1-DOD\right){C}_{bat}$, $SO{C}_{max}={C}_{bat}$, and ${C}_{bat}$ is the nominal battery capacity, DOD is considered 0.80^55^. Charging and discharging efficiencies were set to 85% and 100%, respectively, consistent with literature values. The technical specifications of the selected battery are listed in Table 5. A 12 V, 300 Ah deep-cycle battery was selected because it is commercially available and commonly used in residential stand-alone PV systems. This capacity provides modular storage, allowing the total battery-bank capacity to be adjusted by varying the number of batteries during the optimization process.

**Table 5:** Technical specification of battery^46^.

Type	Photonic Universe
Capacity	300 Ah
Voltage	12 V
Capital cost	700 $/kWh^54^
Replacement cost	500 $/kWh^54^
Lifetime	4 yrs

#### Reliability model

The concept of Loss of Power Supply Probability (LPSP) is applied in this study to evaluate the reliability of the PV-battery system. LPSP is a reliability index expressed as a percentage ranging from 0 to 100%. A value of 0% indicates that the system always satisfies the load (no unmet demand), whereas a value of 100% indicates that the load is completely unmet over the simulation period. Because the system is designed as a stand-alone configuration without grid support, a strict reliability criterion of LPSP = 0% was imposed. In this work, the LPSP is calculated using the following relation^45,56^:

14 $$LPSP=\frac{\sum_{t=1}^{8760}{P}_{loss}(t) }{\sum_{t=1}^{8760}{P}_{L}(t)}\times 100$$

where $\sum_{t=1}^{8760}{P}_{loss}(t)$ is the summation of hourly unmet load over a one-year period (8760 h), and $\sum_{t=1}^{8760}{P}_{L}(t)$ is the total annual household electrical demand. To ensure system reliability, the following constraint is imposed^53^:

15 $$LPSP\le {LPSP}_{required}$$

where LPSP_required_ is the targeted value of LPSP. In this study, it is fixed at 0%, meaning that the entire household load must always be supplied without interruption. The Renewable Contribution (RC) is defined as the complement of LPSP, representing the percentage of the load supplied by renewable energy. It is expressed as:

16 $$RC=\frac{\sum_{t=1}^{8760}\left({P}_{L}(t)-{P}_{loss}(t)\right)}{\sum_{t=1}^{8760}{P}_{L}(t)}\times 100$$

Since LPSP represents the fraction of unmet load, RC can also be expressed as *RC=100-LPSP*. Since the system under consideration is designed to be fully powered by PV with battery storage and constrained to LPSP = 0%, the renewable contribution reaches 100%, ensuring that the household demand is always met using renewable resources without reliance on external backup.

#### Economic model

The economic performance of the PV-battery system is evaluated using the net present cost (NPC) approach. The NPC of each system component is defined as^45^:

17 $$NP{C}_{component}={C}_{capital}+{C}_{replacement}+{C}_{o\$m}$$

where C_capital_ is the initial capital cost of the component (including installation), C_replacement_ is the present value of replacement costs during the project lifetime, and Co&m represents the operation and maintenance (O&M) expenses. Component replacement costs were calculated according to their respective lifetimes (battery: 4 years, inverter: 15 years) within the 25-year project lifetime and included in the NPC evaluation. The LCOE is a fundamental indicator used to assess the average cost of generating one kilowatt-hour (kWh) of electricity from the system. It is expressed as:

18 $$LCOE= \frac{{NPC}_{component} \mathsf{x} CRF}{{E}_{yearly}}$$

here E_yearly​_ is the total annual electrical energy demand (kWh), and NPC_component​_ is the net present cost of all system components. The term CRF denotes the capital recovery factor^46^, which converts the initial investment into equivalent annualized cost and is defined as:

19 $$CRF=\frac{i{\left(1+i\right)}^{N}}{{\left(1+i\right)}^{N}-1}$$

Here, i is the real interest rate (8%) and N is the project lifetime in years considered 25 years^57^.

#### System optimization

The developed techno-economic models were applied to determine the optimal configuration of the PV-battery system under both clean and dusty surface conditions. Two cases were examined: (i) when the PV modules operate with a clean surface, and (ii) when dust accumulation is present with densities of 0.7, 1.4, and 2.1 mg/cm^2^. The optimization objective was to identify the minimum-cost configuration that guarantees 100% renewable contribution and zero loss of power supply probability (LPSP = 0).

The optimization procedure was implemented in MATLAB by varying the number of PV modules and batteries across all feasible configurations. For each configuration, hourly simulations were carried out for a full year (8760 h), considering solar radiation, ambient temperature, load demand, and battery dynamics. The state of charge (SOC) was updated at each step based on the balance between PV generation and load demand. If unmet demand occurred, it was recorded and used to calculate LPSP. Only configurations with LPSP = 0% were retained as feasible.

The optimization does not rely on heuristic or gradient-based algorithms. Instead, an exhaustive enumeration approach was adopted by explicitly varying only two discrete decision variables: the number of PV modules and the number of batteries. The bounds of these variables were selected sufficiently wide to include clearly under-sized, marginally feasible, and over-sized system configurations, ensuring that the minimum-LCOE solution under the LPSP = 0% constraint was not limited by the search space. This approach guarantees that the identified optimal configuration represents the true minimum-cost solution among all fully reliable PV-battery combinations considered.

**Fig. 8:**
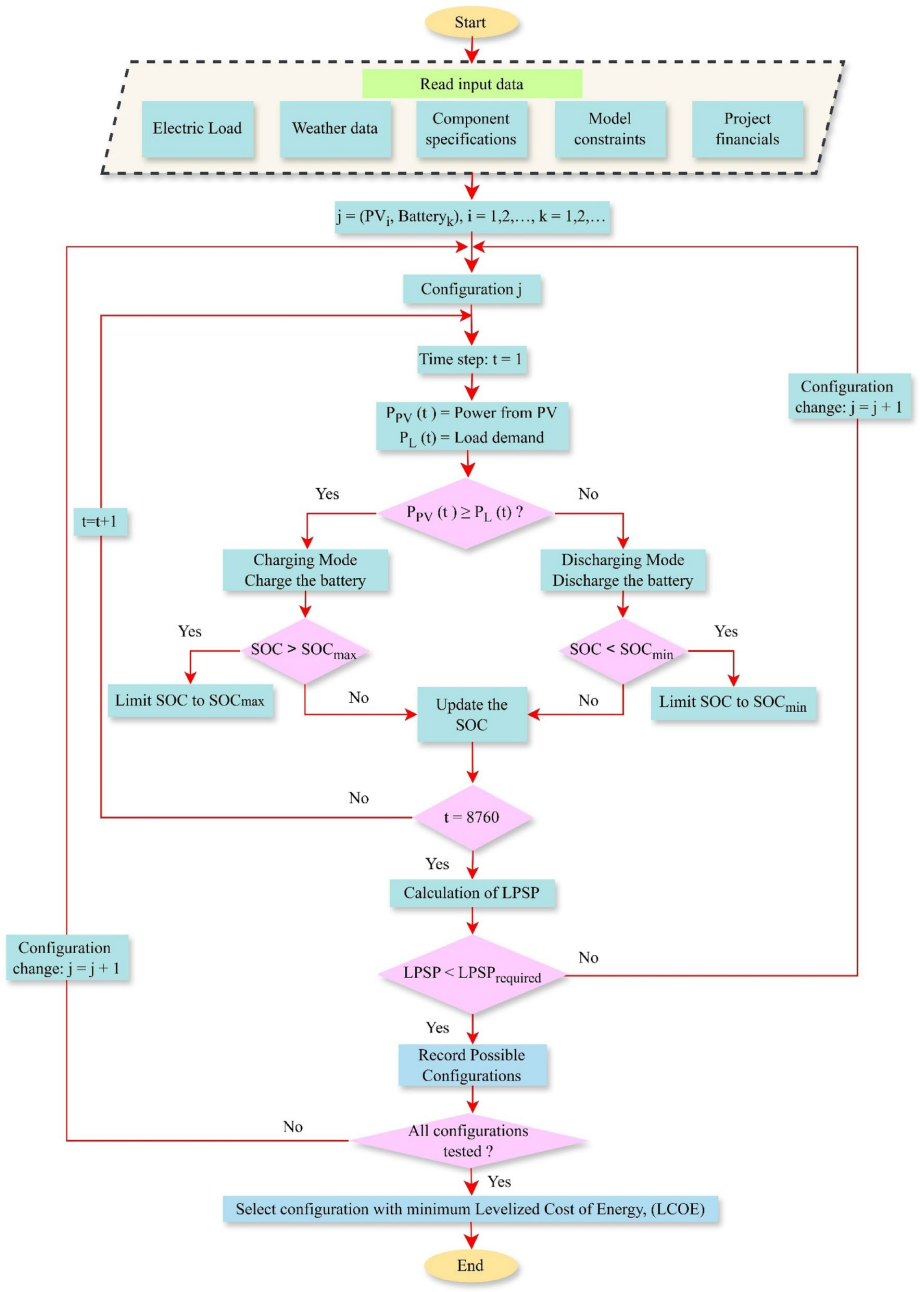
Flowchart of the optimization process for the PV-battery system.

For every feasible case, the net present cost (NPC) was calculated, incorporating capital, replacement, and operation and maintenance (O&M) costs of PV modules, batteries, and the inverter. The LCOE was then computed, and the configuration with the lowest LCOE was selected as the optimal design. The optimization algorithm is illustrated in Fig. 8. The process begins with input data (load profile, weather conditions, component specifications, system constraints, and financial parameters). For each configuration, the PV generation and battery state of charge are simulated, unmet demand is evaluated, and LPSP is calculated. Feasible configurations are recorded, and after all possibilities are tested, the algorithm selects the configuration that yields the minimum LCOE while maintaining full reliability.

## Results and discussion

This section is divided into two main subsections. The first subsection presents the simulation results obtained from the developed MATLAB model, focusing on the optimal sizing of the PV-battery system under clean surface conditions while ensuring full load coverage with renewable energy. The second subsection provides a comparative assessment of the system’s optimal configuration under different dust accumulation levels (0.7, 1.4, and 2.1 mg/cm^2^), highlighting the required increases in PV modules and battery capacity to maintain reliability and achieve the minimum LCOE. To isolate the impact of dust accumulation on system sizing, other design parameters such as PV tilt angle, inverter sizing rules, and dust representation were kept constant following standard design practice. While alternative choices may affect absolute system sizes, they do not alter the comparative trends between clean and dusty scenarios, which is the primary focus of this study.

### Optimal sizing with active clean surface

To establish the baseline performance of the stand-alone PV-battery system, optimization was first conducted under clean surface conditions. This case represents the ideal scenario where dust accumulation on the PV modules is absent, allowing the system to operate at its maximum efficiency. The results provide insight into the required balance between PV generation capacity and battery storage in order to achieve reliable and cost-effective electricity supply for the household demand. Three key performance indicators were evaluated: Loss of Power Supply Probability (LPSP), Renewable Contribution (RC), and the LCOE.

**Fig. 9:**
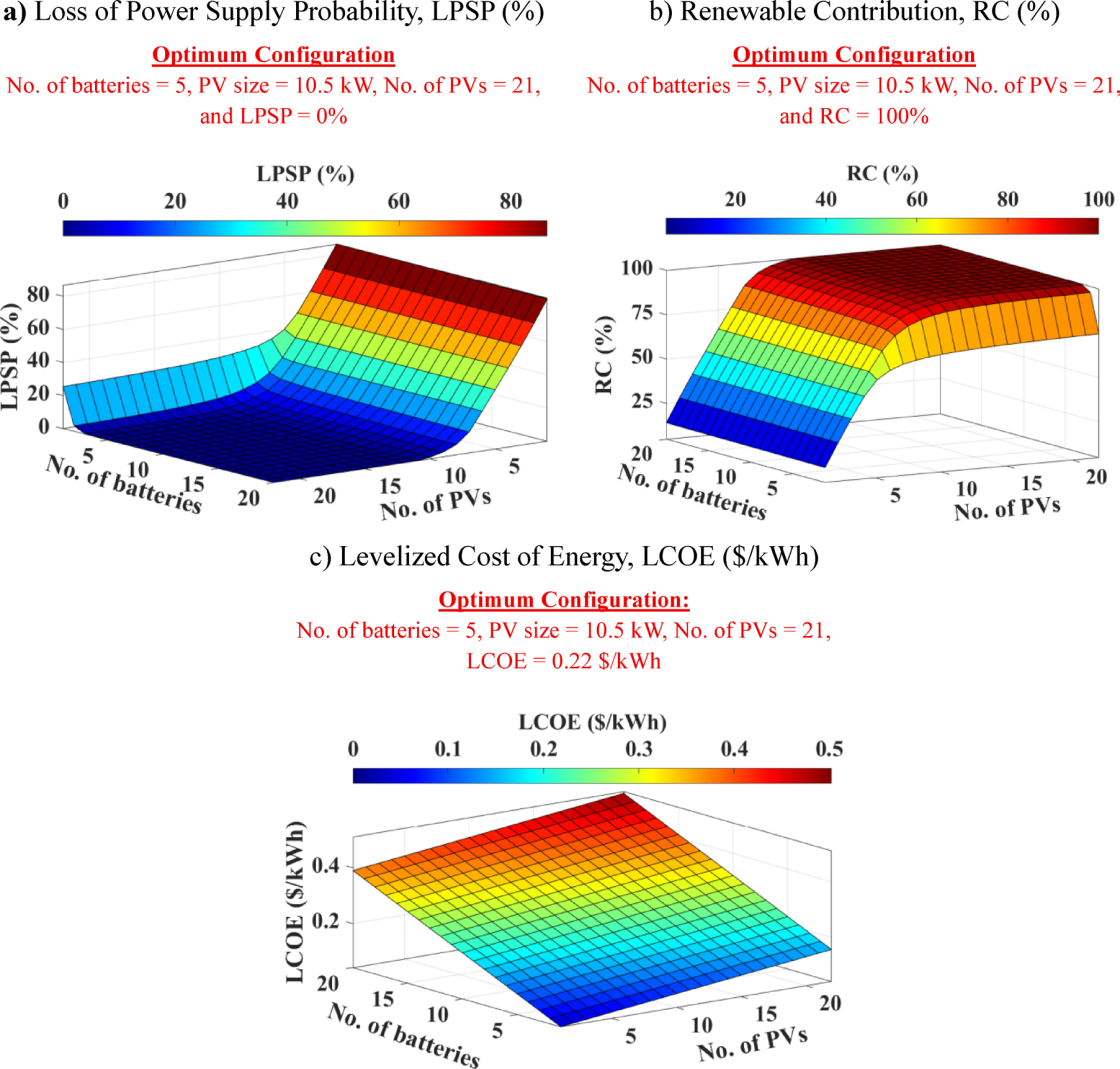
System performance under clean surface conditions: (**a**) loss of power supply probability (LPSP) for different PV-battery configurations, (**b**) renewable contribution (RC) as a function of PV and battery sizes, and (**c**) LCOE for all configurations.

Figure 9a illustrates the variation of LPSP across all simulated configurations of PV modules and batteries. As expected, configurations with very few PV modules and insufficient storage capacity show high values of LPSP, exceeding 80%. This indicates that in such cases, the system frequently fails to meet demand, leaving large portions of the load unmet. However, as the number of PV modules and batteries increases, the LPSP progressively declines. Beyond a certain threshold (approximately 10–12 PV modules combined with 8 or more batteries), the LPSP approaches zero, meaning that the system achieves full reliability and can supply the load continuously without interruptions.

Figure 9b shows the RC of the system for the same range of configurations. The RC represents the percentage of household load supplied entirely by renewable energy. The results mirror the LPSP trend but from the opposite perspective: low-capacity configurations yield RC values below 50%, reflecting insufficient renewable penetration. As both PV and battery capacities increase, the RC rises sharply and eventually stabilizes close to 100%. This confirms that a properly sized PV-battery system under clean conditions can achieve complete renewable coverage of the household demand.

Figure 9c illustrates the variation of the levelized cost of electricity (LCOE) for different PV-battery configurations. The results show a clear increasing trend: as the number of PV modules and batteries rises, the LCOE steadily increases. This occurs because adding more components directly raises the capital and replacement costs of the system. While larger configurations improve reliability, the economic cost per unit of electricity becomes higher since the additional investment is not offset by proportional gains in useful energy output. Therefore, the lowest LCOE values are observed in smaller system sizes, and the cost escalates progressively with system expansion.

**Fig. 10:**
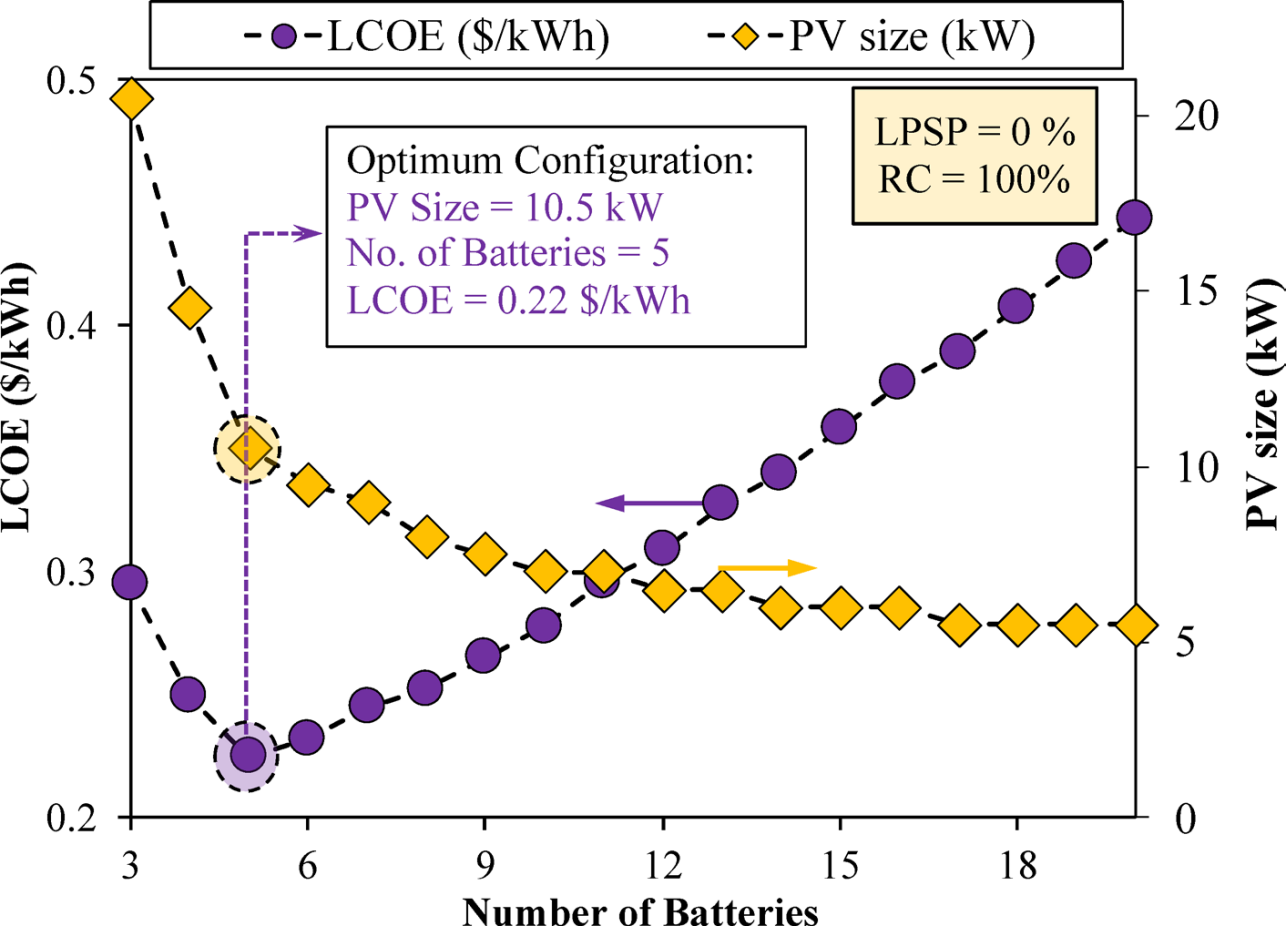
Optimal PV-battery configuration under clean conditions: 10.5 kWp PV and 5 batteries with minimum LCOE of 0.22 $/kWh (LPSP = 0%, RC = 100%).

Figure 10 illustrates the relationship between the number of batteries, PV size, and the resulting Levelized Cost of Electricity (LCOE) for the PV-battery system operating under clean surface conditions, while maintaining full reliability (LPSP = 0%, RC = 100%). The figure presents two key trends. The first trend, shown by the purple curve, represents the variation of LCOE with the number of batteries. At very low storage capacities (3–4 batteries), the LCOE is relatively high (above 0.3 $/kWh) because the system requires oversizing of the PV array to compensate for insufficient storage. As the number of batteries increases, the LCOE decreases sharply, reaching its minimum value when five batteries are installed. Beyond this point, further increases in storage capacity lead to a steady rise in LCOE, since additional batteries increase system cost without yielding proportional improvements in performance.

The second trend, shown by the yellow curve, corresponds to the PV size required to achieve full reliability at different battery numbers. With fewer batteries, a larger PV array is necessary to meet the demand, reaching nearly 20 kWp when only three batteries are used. As storage capacity increases, the required PV size decreases significantly, stabilizing at approximately 10.5 kWp when five batteries are installed. Beyond this configuration, PV size remains relatively constant, indicating that additional batteries do not reduce the required PV capacity but instead raise system cost. As highlighted in the figure, the optimal design consists of 10.5 kWp of PV capacity and 5 batteries, achieving a minimum LCOE of 0.22 $/kWh. The larger PV capacity compared to the household peak load results from the strict LPSP = 0% reliability requirement, where system sizing is governed by annual energy adequacy rather than instantaneous demand. This configuration ensures 100% renewable contribution and uninterrupted power supply (LPSP = 0%), while minimizing the overall cost of electricity production. This result demonstrates the importance of balancing PV generation and storage capacity. Oversizing either component unnecessarily increases cost, while insufficient sizing compromises reliability. The identified optimum thus represents the most cost-effective and technically viable solution under clean surface conditions. Although increasing PV capacity can partially compensate for limited storage, additional PV generation alone cannot fully replace the role of batteries in supplying energy during nighttime and low-irradiance periods. Therefore, reliable stand-alone operation requires an appropriate balance between PV generation and battery storage capacities.

**Fig. 11:**
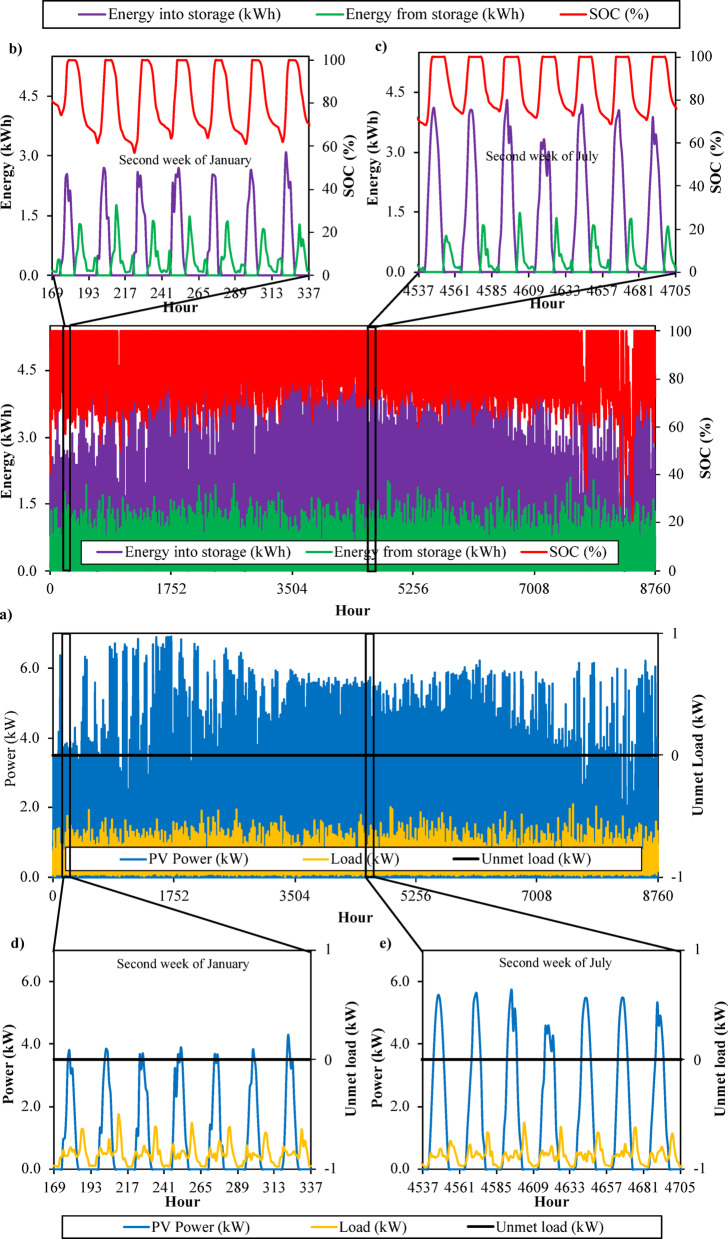
Hourly performance of the optimized stand-alone PV-battery system (10.5 kWp PV, 5 batteries): (**a**) annual energy charged into/out of storage and battery state of charge (SOC), together with PV power, household load, and unmet load; (**b**) storage operation during the second week of January; (**c**) storage operation during the second week of July; (**d**) PV power, load demand, and unmet load during the second week of January; and (**e**) PV power, load demand, and unmet load during the second week of July.

Figure 11a shows the annual performance of the PV-battery system under the optimal configuration (10.5 kWp PV and 5 batteries). The top subplot illustrates the energy flow into and out of the storage system along with the state of charge (SOC). The SOC (red line) fluctuates between ~ 20% and 100%, confirming that the battery operates within its allowable limits. The purple area represents the charging energy supplied to storage, while the green area indicates energy discharged from the batteries to meet the load. These patterns follow the daily solar cycle, with frequent charging during daytime hours and discharging at night. It should be noted that charging and discharging do not occur simultaneously within the same time step. The apparent overlap in the annual plot results from the continuous visualization of hourly data, where charging and discharging occur at different times within the daily cycle.

The bottom subplot presents the PV power generation, household load, and unmet load. The PV output (blue) is consistently higher than the demand (yellow), and the unmet load (black line) remains at zero throughout the year, confirming full reliability (LPSP = 0). The apparent surplus PV generation results from the strict design constraints of LPSP = 0% and RC = 100%, which require sufficient generation during low-irradiance and winter periods; therefore, system sizing is governed by annual reliability requirements rather than instantaneous load magnitude.

Figure 11b zooms into the second week of January (winter season) for the top subplot. During this period, SOC shows daily cycles, starting from ~ 50–60% and recharging close to 100% by midday due to moderate solar availability. Energy charging into storage and discharging from storage alternate consistently, demonstrating the battery’s role in balancing the mismatch between PV generation and load during winter conditions.

Figure 11c presents the same details for the second week of July (summer season). Here, stronger solar radiation results in more pronounced charging cycles, with SOC reaching and maintaining near 100% for extended periods. Charging energy is more dominant compared to winter, and discharging is less pronounced because PV generation is often sufficient to directly meet the demand while still topping up the batteries. This highlights the seasonal variation in system operation, with higher charging activity in summer.

Figure 11d shows the winter week (January) for the bottom subplot (PV power, load, and unmet load). The PV output is consistently higher than the household load, ensuring the load is fully covered even in the colder season. The unmet load (black line) remains flat at zero, indicating that the battery effectively supports demand when PV output is reduced (e.g., during evenings).

Figure 11e depicts the summer week (July) for the bottom subplot. Here, the PV output peaks at higher values compared to winter, reflecting the stronger solar resource. The load profile remains relatively stable, and the unmet load (black) continues to be zero. Surplus PV energy during midday is directed to charge the batteries, ensuring consistent supply during non-solar hours.

### Optimal sizing with active dusty surfaces

**Fig. 12.**
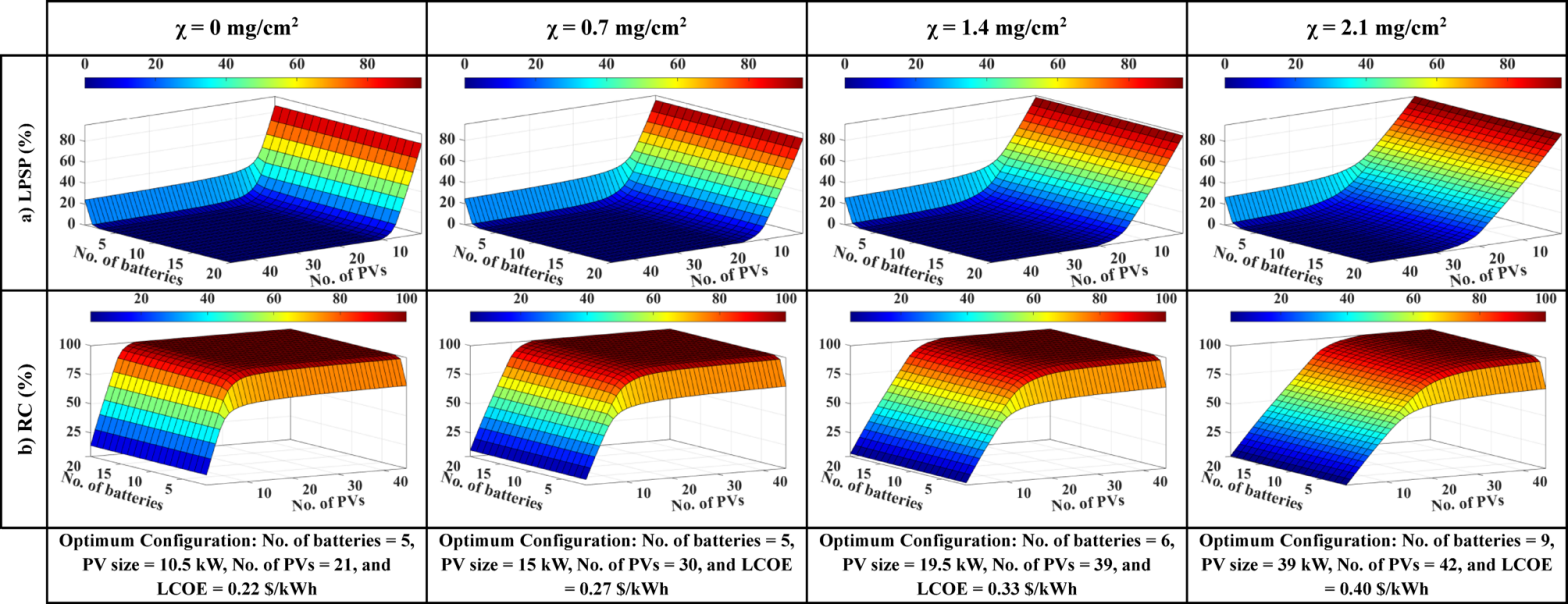
3D surfaces of loss of power supply probability (LPSP) and Renewable Contribution (RC) for PV-battery configurations under different dust accumulation densities (χ = 0, 0.7, 1.4, and 2.1 mg/cm^2^).

Dust deposition on PV modules significantly alters the performance and sizing requirements of PV-battery systems. As dust density increases, the amount of solar radiation reaching the active surface decreases, reducing the effective energy harvested by the system. To maintain the same level of reliability, additional PV modules and storage are often required. It should be noted that increasing PV and battery capacity is not the only possible mitigation approach for dust-related performance degradation. Alternative strategies such as periodic cleaning, anti-soiling coatings, or improved operational control may also reduce soiling losses. However, the present study focuses specifically on system sizing adaptation under fixed operating and maintenance conditions in order to quantify the direct impact of dust accumulation on optimal PV-battery design. Figure 12 presents the 3D surfaces of LPSP and RC for different PV-battery configurations under four dust accumulation densities (χ = 0, 0.7, 1.4, and 2.1 mg/cm^2^). These surfaces provide a comprehensive view of how system reliability and renewable share evolve with increasing dust levels.

For the clean surface case (χ = 0 mg/cm^2^), the LPSP surface declines sharply as PV size and battery number increase, reaching near-zero values with relatively small system sizes. Correspondingly, RC rises steeply and stabilizes close to 100%, showing that high renewable contribution can be achieved without excessive oversizing when dust is absent.

At χ = 0.7 mg/cm^2^, the LPSP surface shifts upward, meaning that smaller PV-battery systems suffer higher power losses compared to the clean case. A larger combination of PVs and batteries is required to push LPSP toward zero. The RC surface also becomes less steep, indicating that higher renewable contribution is only achieved once the PV and battery sizes reach larger values.

With χ = 1.4 mg/cm^2^, the reliability surfaces change more noticeably. The LPSP plane shows a much slower decline with increasing PVs and batteries, and a wider region of configurations experiences high loss of power supply. Similarly, the RC surface plateaus later, demonstrating that only relatively large systems can reach full renewable contribution.

Finally, under the highest dust density (χ = 2.1 mg/cm^2^), the LPSP surface is elevated across almost all configurations, with many smaller and mid-sized systems unable to maintain supply. The RC surface shows that renewable contribution improves only gradually with additional PVs and batteries, reaching high values only in the upper ranges of system capacity.

Figuers 13a, b present the impact of dust accumulation on the LCOE and PV size requirements as a function of the number of batteries, under the conditions of LPSP = 0% and RC = 100%. The results show a consistent trend where dust accumulation significantly increases both system size and cost, shifting the curves upward compared to the clean surface case.

**Fig. 13:**
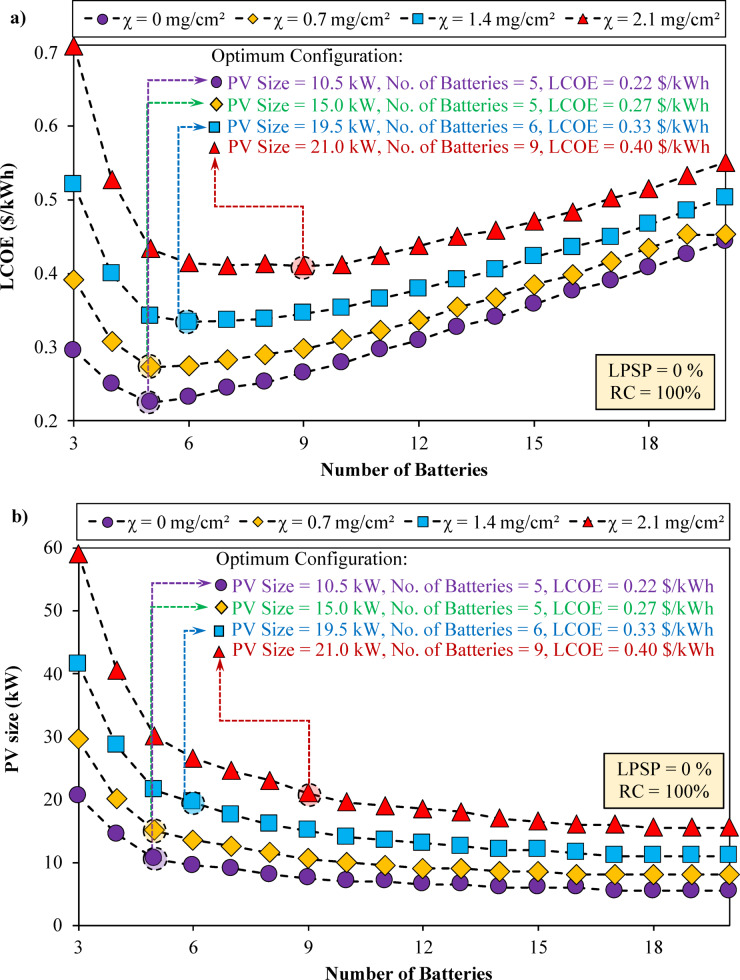
Variation of (**a**) LCOE and (**b**) PV size with number of batteries under different dust accumulation densities (χ = 0, 0.7, 1.4, and 2.1 mg/cm^2^), ensuring LPSP = 0% and RC = 100%.

It should be noted that the total annual energy supplied to the load remains nearly constant across all dust scenarios due to the imposed constraint of LPSP = 0%. As dust accumulation reduces PV energy output, the optimization compensates by increasing the PV capacity and, in some cases, battery storage. Therefore, the impact of dust is not observed as a reduction in delivered energy, but rather as an increase in system size and cost required to maintain full reliability. This highlights that neglecting dust effects during system design can lead to underestimation of required capacity rather than immediate energy shortfall.

For the clean surface condition (χ = 0 mg/cm^2^), the system achieves its best performance with an optimal configuration of 10.5 kWp PV capacity and 5 batteries, resulting in a minimum LCOE of 0.22 $/kWh. The LCOE curve shows a U-shaped behavior: too few batteries raise costs due to unmet storage needs, while too many batteries increase costs without proportional benefits. Correspondingly, the PV size curve stabilizes once adequate storage is available, showing no need for further oversizing.

When dust increases to χ = 0.7 mg/cm^2^, the system requires 15 kWp PV capacity with 5 batteries to maintain reliability, raising the LCOE to 0.27 $/kWh. This demonstrates that even moderate dust reduces solar transmittance enough to necessitate additional PV modules, while battery requirements remain relatively stable. At χ = 1.4 mg/cm^2^, the effect of dust becomes more pronounced. The system requires 19.5 kWp of PV capacity and 6 batteries, with the LCOE rising to 0.33 $/kWh. Here, both generation and storage need to be expanded, reflecting the compounding influence of higher dust levels. Finally, under the heaviest dust scenario (χ = 2.1 mg/cm^2^), the system demands significant oversizing: 21 kWp PV capacity and 9 batteries, resulting in an LCOE of 0.40 $/kWh. This value is nearly double that of the clean-surface case, emphasizing the severe economic and technical penalties of high dust accumulation. Overall, Figs. 13a, b highlight that dust deposition drives up both PV size and LCOE, with storage playing a secondary but supportive role. The findings stress the necessity of considering dust impacts in PV-battery system design for arid and semi-arid regions.

**Fig. 14:**
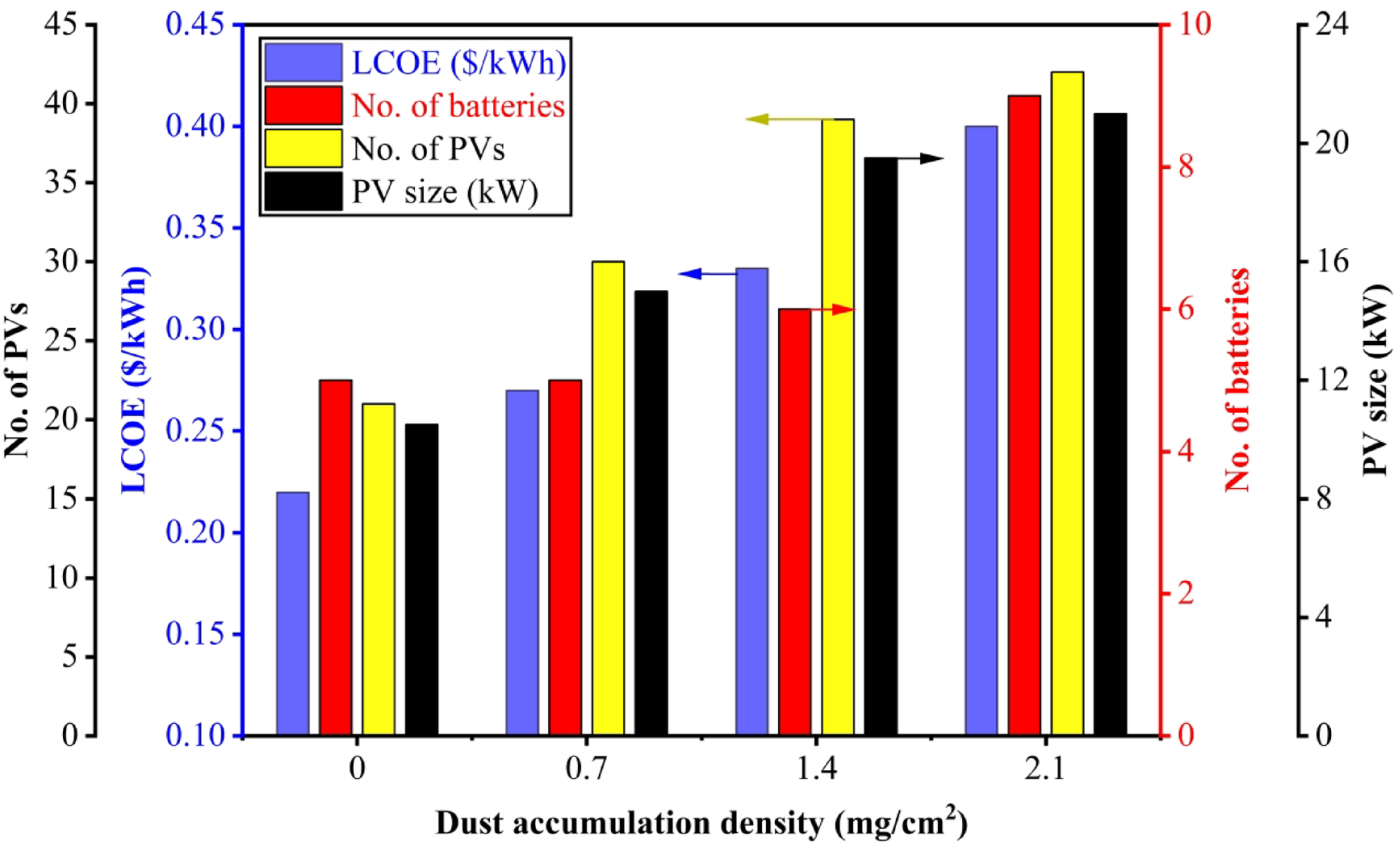
Comparison of optimal PV-battery configurations under different dust accumulation densities (χ = 0, 0.7, 1.4, and 2.1 mg/cm^2^) showing variations in LCOE, PV size, number of modules, and number of batteries.

Figure 14 summarizes the effect of dust accumulation on the optimal configuration of the PV-battery system, showing how LCOE, PV size, number of modules, and battery requirements evolve with increasing dust density (χ = 0, 0.7, 1.4, and 2.1 mg/cm^2^). Under clean surface conditions (χ = 0 mg/cm^2^), the system achieves its most favorable performance with an LCOE of 0.22 $/kWh, supported by a PV capacity of 10.5 kWp (21 modules) and 5 batteries. When dust density rises to χ = 0.7 mg/cm^2^, the LCOE increases to 0.27 $/kWh, reflecting a 22.7% rise compared to the clean case. To maintain full reliability (LPSP = 0%, RC = 100%), the PV size must be expanded to 15 kWp (30 modules), a 43% increase, while the battery requirement remains unchanged.

As dust accumulation intensifies, the system requires progressively larger capacities. At χ = 1.4 mg/cm^2^, the LCOE grows to 0.33 $/kWh, nearly 50% higher than the baseline. The PV size increases to 19.5 kWp (39 modules), representing an 86% rise, while the battery count climbs to 6, about 20% more than the clean-surface case. Under the most severe dust density (χ = 2.1 mg/cm^2^), the oversizing becomes substantial, with PV size reaching 21 kWp (42 modules), doubling compared to the dust-free condition. The number of batteries escalates to 9, an 80% increase, and the LCOE reaches 0.40 $/kWh, about 82% higher than the clean case. These results clearly demonstrate that dust accumulation imposes significant penalties on system design, forcing oversizing of both PV and storage while sharply rising costs.

The optimal clean-surface configuration obtained in this study (10.5 kWp PV and 5 batteries) is consistent with PV-battery system sizes reported in the literature using established tools such as HOMER for similar residential load profiles and climatic conditions. This agreement provides confidence in the developed MATLAB framework, while allowing the direct integration of experimentally derived dust transmittance effects that are not readily available in standard optimization tools.

## Conclusion

This study investigated the impact of dust accumulation on the optimal sizing of a PV-battery system for a typical household in Saudi Arabia, aiming for 0% Loss of Power Supply Probability (LPSP) and 100% Renewable Contribution (RC) at the lowest possible LCOE. Dust accumulation densities of χ = 0, 0.7, 1.4, and 2.1 mg/cm^2^ were considered, combining experimental characterization of dust samples with techno-economic system modeling. The main findings are summarized below:

- SEM and EDS analyses revealed that dust particles exhibit highly irregular morphologies, ranging from spherical to fibrous, flake-like, and sharp-edged forms, with sizes from a few micrometers up to about ~ 100 µm (average 50 µm). The dust was predominantly composed of oxygen (58.6%) and silicon (11.8%), along with calcium, magnesium, sulfur, aluminum, and trace elements, confirming a dominance of oxides and silicate compounds that strongly influence soiling.
- The optical performance of PV modules was severely affected by dust deposition. Transmittance (τ_dusty_) decreased from 1 (clean surface) to 0.704, 0.496, and 0.349 for χ = 0.7, 1.4, and 2.1 mg/cm^2^, respectively, indicating that even moderate dust levels can reduce incident light by more than 50%.
- Under clean conditions (χ = 0 mg/cm^2^), the optimum configuration was 10.5 kWp PV system (21 modules) and 5 batteries, yielding an LCOE of 0.22 $/kWh.
- With increasing dust density, system oversizing became necessary to preserve reliability. At χ = 0.7, 1.4, and 2.1 mg/cm^2^, the optimum PV capacities rose to 15.0, 19.5, and 21 kW, while batteries increased to 5, 6, and 9 units, respectively.
- The economic penalty was substantial: LCOE rose from 0.22 $/kWh (clean) to 0.27, 0.33, and 0.40 $/kWh under dusty conditions, representing increases of approximately 23%, 50%, and 82%.

Overall, the results demonstrate that dust accumulation significantly alters both the technical sizing and economic performance of PV-battery systems. In arid and semi-arid regions, failure to account for dust effects leads to underestimation of PV and storage requirements, compromising reliability and inflating costs. Future work should focus on dust-mitigation solutions, such as self-cleaning coatings, optimized cleaning schedules, or predictive maintenance strategies, to reduce oversizing requirements and ensure the long-term sustainability of PV-based energy systems in dusty environments.

## Data Availability

The datasets generated and analysed during the current study are available from the corresponding author on reasonable request.

## List of symbols

A: PV module surface area (m^2^)
α_cell_: PV cell absorptivity
β: Tilt angle (°)
β_ref_: Temperature coefficient of maximum power of the PV module (1/°C)
C_bat_: Nominal battery capacity (kWh)
C_capital_: Capital cost of component ($)
C_o&m_: Operation and maintenance cost ($)
C_replacement_: Replacement cost of component ($)
CRF: Capital recovery factor
δ: Solar declination angle (°)
DOD: Depth of discharge (%)
γ: Dust particle optical transmittance factor
Gsc: Solar constant (1367 W/m^2^)
I_b_: Beam radiation on a horizontal surface (W/m^2^)
I_d_: Diffuse radiation on a horizontal surface (W/m^2^)
I: Global radiation on a horizontal surface (W/m^2^)
I_o_: Extraterrestrial solar radiation (W/m^2^)
I_T_: Solar radiation on tilted surface (W/m^2^)
k_T_: Clearness index
LCOE: Levelized cost of electricity ($/kWh)
LPSP: Loss of power supply probability (%)
M_tot_: Total dust mass on surface (g)
NPC: Net present cost ($)
n: Day of the year
P: PV output power (W)
P_c_(t): Converter output power at hour *t* (kW)
P_L_(t): Load demand at hour *t* (kW)
P_Demand_(t): Hourly power demand (kW)
P_PV_(t): Hourly PV output power (kW)
E_yearly_: Total annual electrical load demand (kWh)
ρ: Dust particle density (g/cm^3^)
ρ_g_: Ground reflectance (dimensionless)
R_b_: Geometric factor (ratio of beam radiation)
R_d_: Dust particle radius (µm)
RC: Renewable contribution (%)
SOC(t): Battery state of charge at hour *t* (kWh)
SOC_max_: Maximum allowable state of charge (kWh)
SOC_min_: Minimum allowable state of charge (kWh)
τ_clean_: Transmittance of clean PV glass
τ_dusty_: Transmittance of dusty PV glass
τ_g_: Glass cover transmissivity
η: Converter efficiency
η_chg_: Battery charging efficiency
η_dis_: Battery discharging efficiency
η_el_: PV electrical efficiency
η_ref_: Module efficiency at STC
